# Renal Denervation for Resistant Hypertension in the contemporary era: A Systematic Review and Meta-analysis

**DOI:** 10.1038/s41598-019-42695-9

**Published:** 2019-04-17

**Authors:** Pradyumna Agasthi, Justin Shipman, Reza Arsanjani, Moses Ashukem, Marlene. E. Girardo, Charan Yerasi, Nithin. R. Venepally, Floyd David Fortuin, Farouk Mookadam

**Affiliations:** 1 Division of Cardiovascular Diseases, Mayo Clinic Arizona, Phoenix, Arizona USA; 2 Division of Cardiovascular Medicine, University of Arizona, Phoenix, Arizona USA; 3 Department of Health Sciences Research, Mayo Clinic Arizona, Scottsdale, Arizona USA; 4 Department of Cardiovascular Diseases, St. Joseph’s Hospital and Medical Center, Phoenix, Arizona USA

**Keywords:** Interventional cardiology, Hypertension

## Abstract

Renal denervation (RDN) is a catheter-based ablation procedure designed to treat resistant hypertension (RH). The objective of our study is to determine the effect of RDN on blood pressure and renal function in patients with RH in comparison to medical therapy alone. We performed an extensive literature search for randomized control trials (RCT) reporting office and 24 hr. blood pressure changes and estimated glomerular filtration rate (eGFR) at baseline and 6 months. We calculated a weighted standardized mean difference of blood pressure and renal outcomes between RDN and control groups using random effects models. Our search yielded 608 studies of which we included 15 studies for the final analysis. A total of 857 patients were treated with RDN and 616 patients treated with medical therapy ± sham procedure. Only 5 studies were double-blinded RCT with sham control. The adjusted standardized mean difference in the change in office based systolic and diastolic pressures (p = 0.18; p = 0.14); 24 hr. systolic and diastolic pressures (p = 0.20; p = 0.18); and eGFR (p = 0.20) from baseline to 6 months is statistically insignificant with significant heterogeneity. Subgroup analysis showed that among sham controlled trials, 24 hr. systolic blood pressure showed a modest but statistically significant benefit favoring renal denervation in patients with RH. Our meta-analysis of 15 RCTs showed no significant benefit of RDN on blood pressure control in patients with resistant hypertension. Subgroup analysis of sham control studies showed a modest benefit in 24 hr. systolic blood pressure at 6 months with RDN.

## Introduction

Resistant hypertension (RH) is defined as blood pressure that remains above guideline-directed goal despite the concurrent use of at least three antihypertensive agents of different classes, one of which is a diuretic^1^. The prevalence of RH based on recent studies is ranging from as low as 12.8%^2^, while others have shown RH to be up to 25–30% in the general hypertensive population^3^. A 2012 study by Dougherty *et al*. showed the incidence of resistant hypertension to be 1.9% within 1.5 years of newly diagnosed hypertensive patients undergoing treatment^4^.

When hypertension remains uncontrolled with lifestyle changes and antihypertensive medications, renal denervation has been a proposed intervention to aid in the treatment of RH since it was first used a decade ago^5^. Since then, there have been numerous studies and randomized control trials to evaluate the effectiveness of this procedure in treating RH with variable results.

One of the most recent meta-analysis w by Fadl Elmula *et al*. did not show any significant effect on blood pressure in patients with RH following renal denervation^6^. Over the past year, multiple randomized control trials evaluating the effect of renal denervation on RH showed promising results including: WAVE-IV^7^, SPYRAL HTN-ON MED^8^, INSPiRED^9^, and a Polish study by Warchol-Celinska *et al*.^10^ We, therefore, conducted a systematic review and meta-analysis based on randomized control trials to assess the efficacy of renal denervation in patients with RH at 6 months in comparison to medical therapy alone ± sham procedure.

## Results

**Figure 1:**
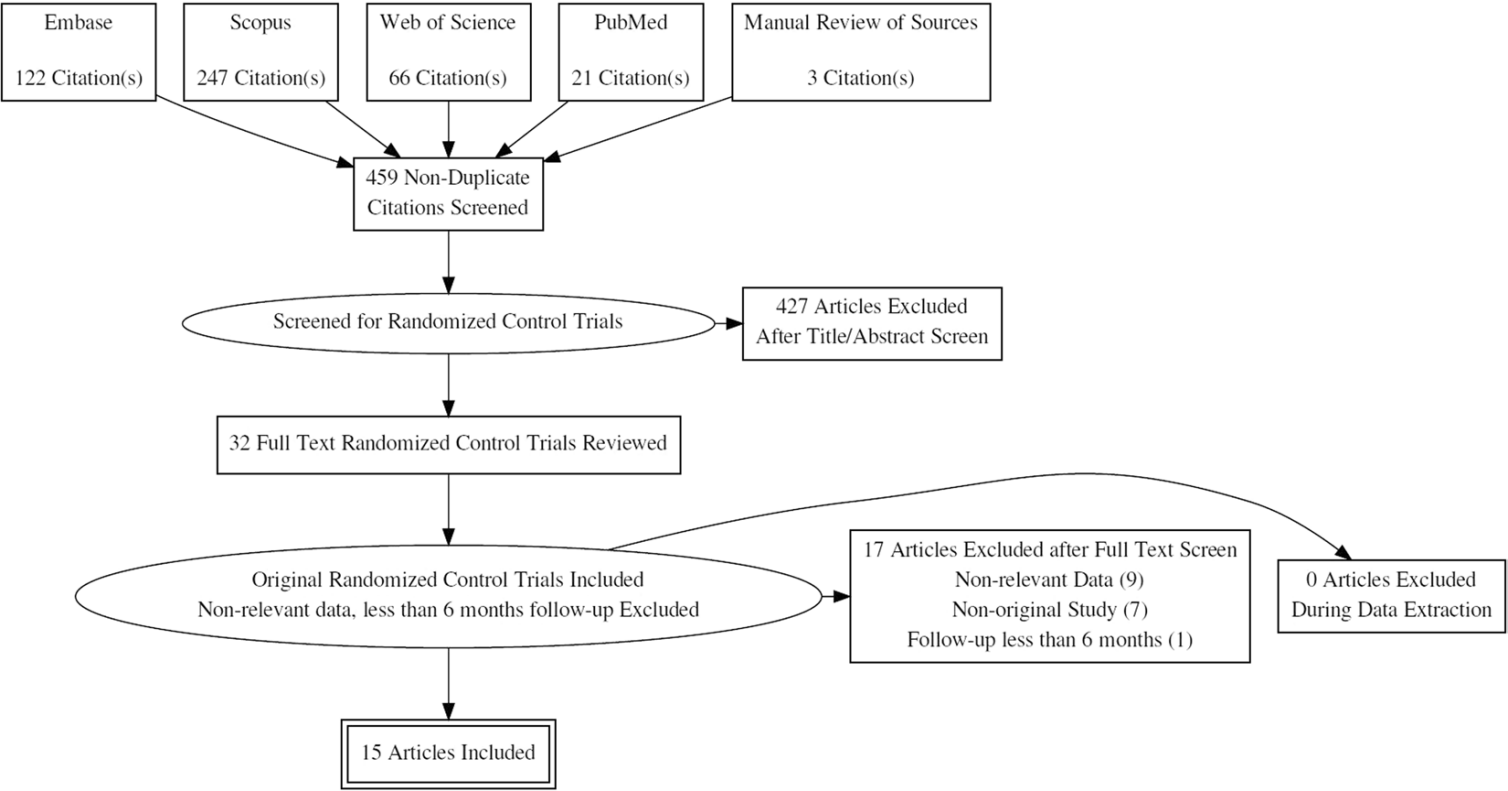
PRISMA Flow Chart.

Our search yielded 608 studies. After exclusion of duplicates, 456 studies remained of which we included 15 studies for the final analysis (Fig. 1). A total of 857 patients were treated with renal denervation and 616 patients treated with medical therapy ± sham procedure. Of the included studies, only 5 were double blinded randomized control trials with sham control^7,8,11–13^, while the remaining 10 trials were open-label clinical trials with a control group treated with optimal medical therapy^9,10,14–21^. Radiofrequency (RF) ablation was performed in all trials except the WAVE-IV^7^, where ultrasound energy based (US) ablation was performed. Only 13 studies reported a mean change in office based systolic and diastolic blood pressures, and 14 studies reported a mean change in 24 hr. systolic and diastolic blood pressure between baseline and 6 months post-randomization. Only 11 studies reported a change in estimated glomerular filtration rate (eGFR) between baseline and 6 months post-randomization in the treatment and control groups. The baseline characteristics of the included studies are included in Table 1. Baseline characteristics of study participants of the included studies are reported in the Supplementary Tables S1 and S2. The overall quality of the included randomized control trials was high based on the Jadad scale except for the study by Warchol-Celinska *et al*.^10^ which was a low-quality study (Supplementary Table S3).

**Table 1:** Design features of controlled trials included in the meta-analysis.

Author	study acronym	year of publication	Location	Study Design	Total sample size	N (RDN)	N (control)	Renal denervation group	Control group	Drug adherence assessment	Catheter	Renal Denervation Ablation
Esler *et al*.^16^	Symplicity HTN 2	2010	Europe, Australia	Open label multicenter trial	82	49	35	Renal Denervation + Medical therapy	Medical Therapy	Diary	Symplicity	Radiofrequency
Bhatt *et al*.^11^	Symplicity HTN 3	2014	United states	Double blind multicenter trial	535	364	171	Renal Denervation + Medical therapy	Sham + Medical Therapy	Diary	Symplicity	Radiofrequency
Fadl Elmula *et al*.^17^	OSLO RDN	2014	Europe	Open label single center trial	19	9	10	Renal Denervation + Medical therapy	Medical Therapy	Witnessed Medication Intake	Symplicity	Radiofrequency
Rosa *et al*.^20^	PRAGUE-15	2015	Europe	Open label multicenter trial	106	52	54	Renal Denervation + Medical therapy	Medical Therapy (including Spironolactone)	Plasma Drug Concentration	Symplicity	Radiofrequency
Azizi *et al*.^14^	DENERHTN	2015	Europe	Open label multicenter trial	96	48	48	Renal Denervation + Medical therapy	Medical Therapy	Morisky Medication Adherence Scale	Symplicity	Radiofrequency
Desch *et al*.^12^	Symplicity-Flex	2015	Europe	Double blind single center trial	71	32	35	Renal Denervation + Medical therapy	Sham + Medical Therapy	Interview	Symplicity Flex	Radiofrequency
Schneider *et al*.^21^	ISAR-denerve	2015	Europe	Open label single center trial	18	9	9	Renal Denervation + Medical therapy	Medical Therapy	Logbook	Symplicity Flex	Radiofrequency
Kario *et al*.^18^	Symplicity HTN-Japan	2015	Asia	Open label multicenter trial	41	22	19	Renal Denervation + Medical therapy	Medical Therapy	Diary	Symplicity	Radiofrequency
Mathiassen *et al*.^13^	ReSET	2016	Europe	Double blind single center trial	69	36	33	Renal Denervation + Medical therapy	Sham + Medical Therapy	Diary	Symplicity	radiofrequency
Oliveras *et al*.^19^	DENERVHTA	2016	Europe	Open label multicenter trial	27	11	13	Renal Denervation + Medical therapy	Medical Therapy (including Spironolactone)	Haynes-Sackett Test	Symplicity	Radiofrequency
de Jager *et al*.^15^	Sympathy	2017	Europe	Open label multicenter trial	139	95	44	Renal Denervation + Medical therapy	Medical Therapy	Plasma Drug Concentration	Symplicity	Radiofrequency
Jacobs *et al*.^9^	INSPiRED	2017	Europe	Open label multicenter trial	15	6	9	Renal Denervation + Medical therapy	Medical Therapy	Morisky Medication Adherence Scale	EnligHTN	Radiofrequency
Schmeider *et al*.^7^	WAVE-IV	2017	Europe	Double blind multicenter trial	81	42	39	Renal Denervation + Medical therapy	Medical Therapy	Urine Toxicology Analysis	Surround Sound System	Ultrasound
Warchol-Celinska *et al*.^10^	No Acronym	2018	Europe	Open label single center trial	60	30	30	Renal Denervation + Medical therapy	Medical Therapy	Morisky Medication Adherence Scale	Symplicity	Radiofrequency
Kandazari *et al*.^8^	SPYRAL HTN-ON MED	2018	North America, Europe, Australia, Asia	Double blind multicenter trial	80	38	42	Renal Denervation + Medical therapy	Sham + Medical Therapy	Plasma Drug Concentration	Symplicity Spyral	Radiofrequency*

RDN: Renal denervation.

*Renal Denervation performed in the main renal artery + distal branches of renal artery.

The change in blood pressure (24 hr. and office) at 6 months and renal function in patients in the renal denervation (RDN) and the control group for each study is listed in Supplementary Table S4. In the pooled analysis, the unadjusted change in office blood pressure (mmHg) in the RDN group was −14.65 ± 22.29 (systolic) and −6.88 ± 11.89 (diastolic) vs −11.63 ± 22.30 (systolic) and −4.94 ± 11.97 in the control group. The unadjusted change in 24 hr. blood pressure (mmHg) in the RDN group was −7.53 ± 15.14 (systolic) and −4.64 ± 9.18 (diastolic) vs −5.72 ± 15.12 (systolic) vs −3.71 ± 8.95 (diastolic) in the control group. The unadjusted pooled change in eGFR (mL/min/1.73 m^2^) at 6 months in the RDN group was −1.7 ± 11.75 vs −2.39 ± 11.67 in the control group.

### Efficacy of renal denervation on systolic blood pressure

**Figure 2:**
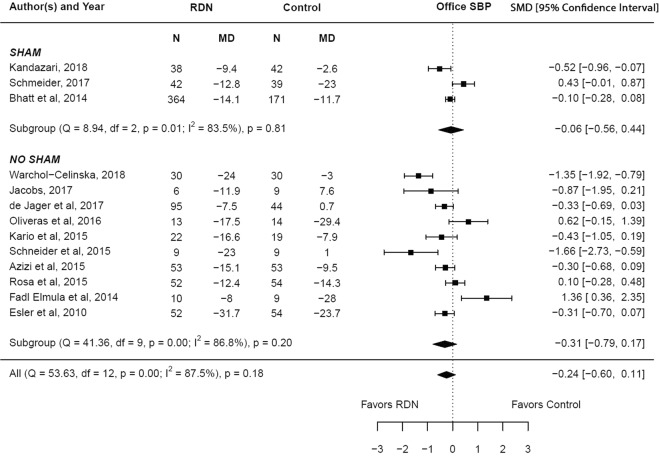
Adjusted standardized mean difference in office systolic blood pressure after renal denervation.

The adjusted standardized mean difference in the change in systolic blood pressure (office) at 6 months between RDN and control groups was −0.24 (95% confidence interval (CI) −0.60–0.11, p = 0.18) which is statistically insignificant. Subgroup analysis showed no statistical significance in both the sham controlled (p = 0.81) and non-sham controlled studies (p = 0.20). There was a high degree of heterogeneity noted among the studies (I^2^ = 87.5%). The results are summarized in Fig. 2. Meta regression analysis showed that underlying coronary artery disease (β = −0.02, p = 0.02) influenced the primary outcome (Supplementary Table S5).

**Figure 3:**
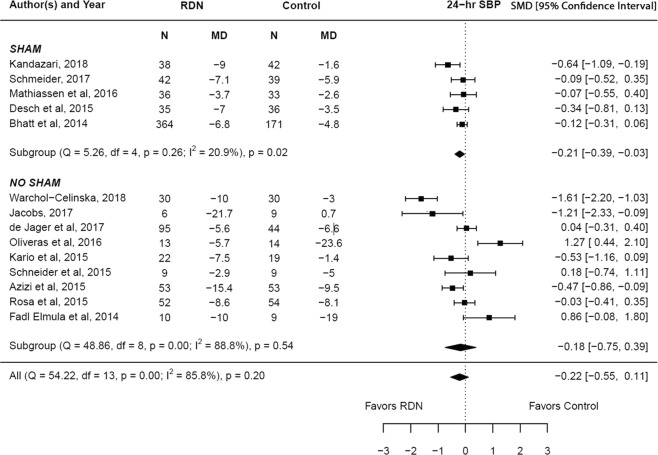
Adjusted standardized mean difference in 24 hr. systolic blood pressure after renal denervation.

The adjusted standardized mean difference in the change in systolic blood pressure (24 hr.) at 6 months between RDN and control groups was −0.22 (95% CI −0.55–0.11, p = 0.20), which was statistically insignificant. However, subgroup analysis among sham control studies showed statistical significance favoring renal denervation (p = 0.02). A high degree of heterogeneity was noted among the studies included in the main analysis (I^2^ = 85.8%). The results are summarized in Fig. 3. However, the subgroup of sham control studies had a low degree of heterogeneity, which likely contributed to the statistical significance achieved in this subgroup. Meta-regression analysis showed that underlying coronary artery disease (β = −0.034; p = 0.001) significantly influenced the change in 24 hr. systolic blood pressure reported by the studies, suggesting that the lower prevalence of coronary artery disease positively affected the reduction in 24 hr. systolic blood pressure (Supplementary Table S5).

### Efficacy of Renal denervation on diastolic blood pressure

**Figure 4:**
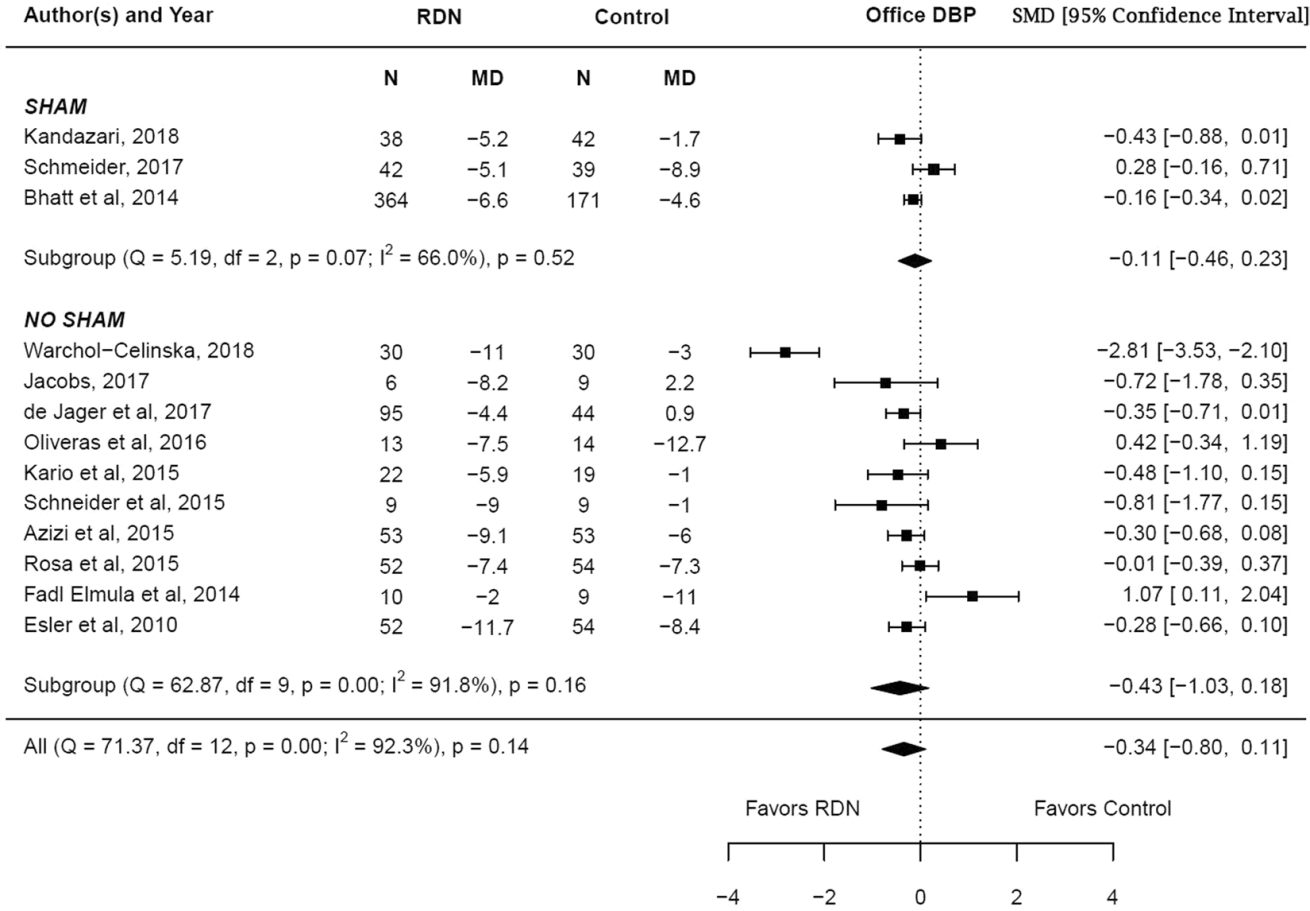
Adjusted standardized mean difference in office diastolic blood pressure after renal denervation.

The adjusted standardized mean difference in the change in diastolic blood pressure (office) at 6 months between RDN and control groups was −0.34 (95% CI −0.80–0.11, p = 0.14) which was not statistically significant. Subgroup analysis showed no difference in both the sham controlled (p = 0.52) and non-sham controlled studies (p = 0.16). There was a high degree of heterogeneity noted among the studies (I^2^ = 92.3%), which likely accounted for the lack of difference noted between both groups. The results are summarized in Fig. 4. Meta regression analysis showed that underlying coronary artery disease (β = −0.03, p = 0.02) and sham controlled study (β = 1.23, p = 0.01) influenced the change in diastolic blood pressure (office) reported by the studies (Supplementary Table S5). This suggests that the lower prevalence of coronary artery disease and sham control study design contributed to the lack of difference noted between both groups.

**Figure 5:**
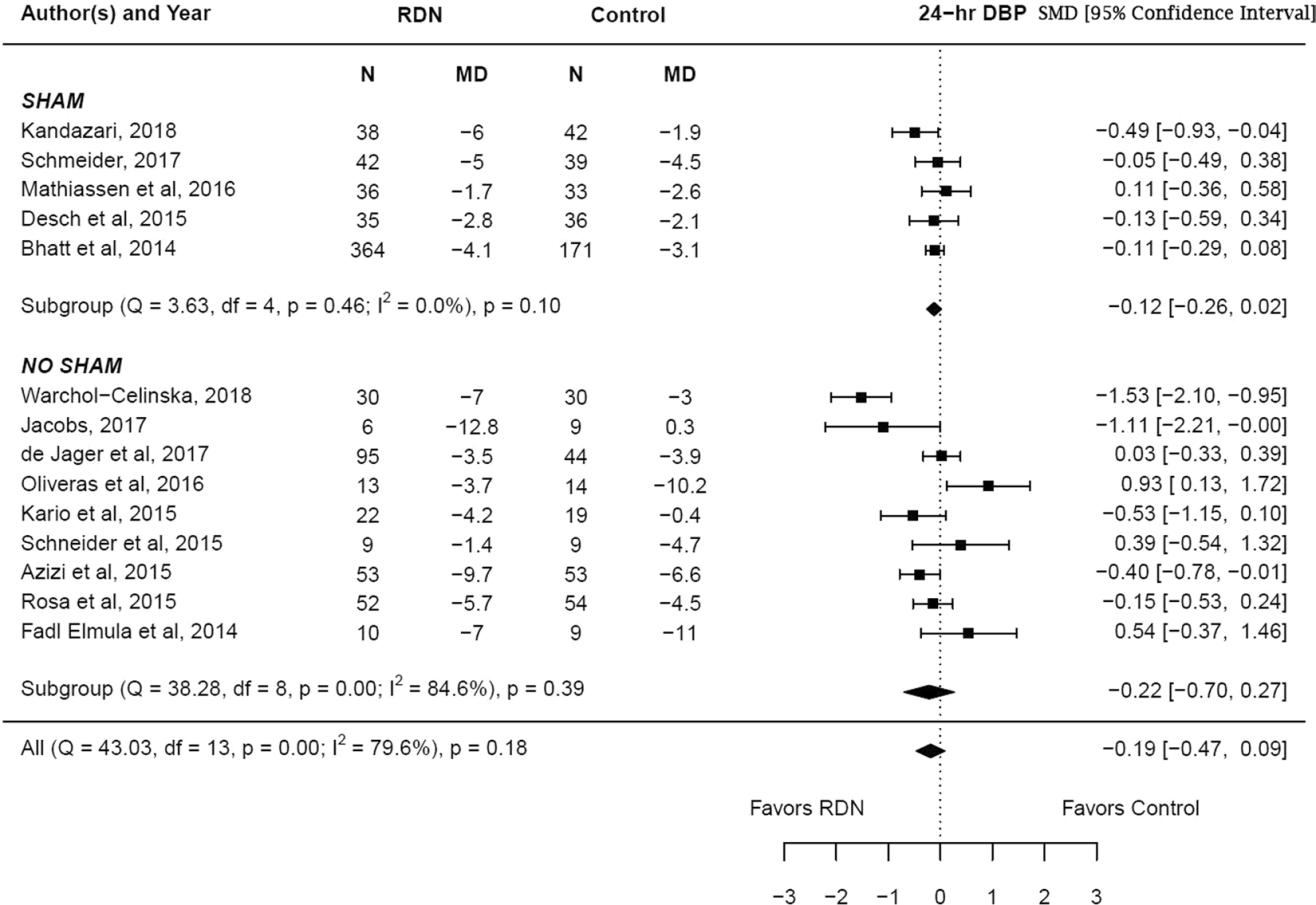
Adjusted standardized mean difference in 24 diastolic blood pressure after renal denervation.

The adjusted standardized mean difference in the change in diastolic blood pressure (24 hr.) at 6 months between RDN and control groups was −0.19 (95% CI −0.47–0.09, p = 0.18) which was not statistically significant. Subgroup analysis showed no statistical significance in both the sham controlled (p = 0.10) and non-sham controlled studies (p = 0.18). There was a high degree of heterogeneity noted among the studies (I^2^ = 79.6%). The sham control studies showed no heterogeneity (I^2^ = 0%), however, the result remained unchanged. The results are summarized in Fig. 5. Meta regression analysis showed that underlying coronary artery disease (β = −0.03, p = 0.005) influenced the change in 24 hr. diastolic blood pressure reported by the studies (Supplementary Table S5).

### Effect of renal denervation on renal function (eGFR)

**Figure 6:**
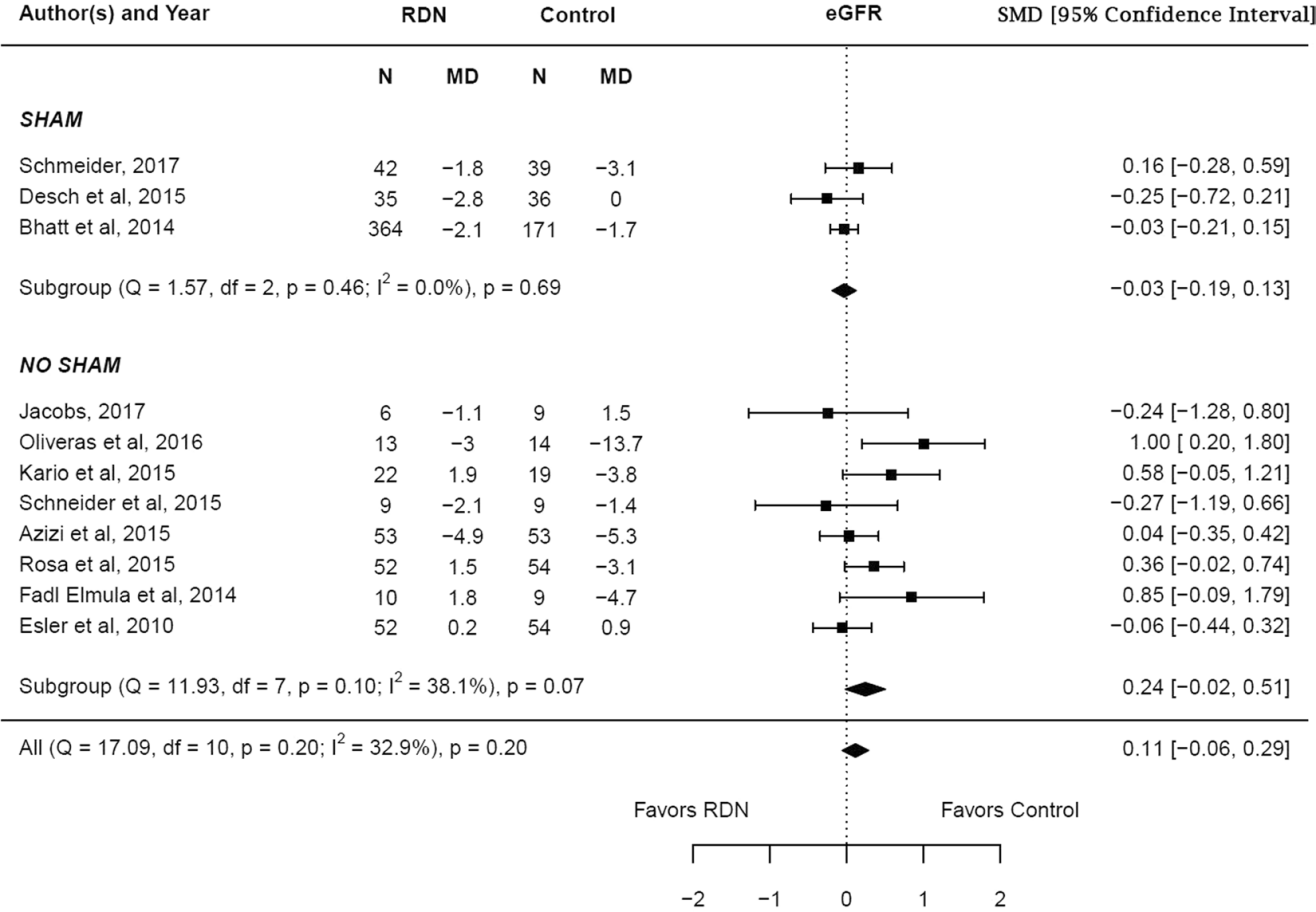
Adjusted standardized mean difference in estimated glomerular filtration rate after renal denervation.

The adjusted standardized mean difference in the change in eGFR at 6 months between RDN and control groups was 0.11 (95% CI −0.06–0.29, p = 0.20) which was statistically insignificant. Subgroup analysis showed no statistical significance in both the sham controlled (p = 0.69) and non-sham controlled studies (p = 0.07). There was a low degree of heterogeneity noted among the studies (I^2^ = 32.9%). The results are summarized in Fig. 6. Meta regression analysis showed that none of the tested covariates influenced the change in eGFR reported by the studies (Supplementary Table S5).

Risk of bias across individual studies was estimated for the five outcomes using Deek’s funnel plot. The funnel plot for the meta-analysis showed significant asymmetry suggesting publication bias. However, the trim-and-fill method showed no need for adjusting the effect size for the meta-analysis.

## Discussion

Our meta-analysis of high-quality randomized control trials (Table S3) shows no significant benefit of RDN over medical therapy in reducing blood pressure in patients with RH. There was however a modest benefit in 24 hr. systolic blood pressure reduction noted with RDN in a subgroup of sham controlled randomized control trials.

Sham controlled randomized control trials have a semi-blinded design which is aimed to negate patient and physician-related confounders on the results as described previously in the literature^22–24^. A Sham procedure typically involves an invasive procedure performed in the patients randomized to the control group. However, perfect blinding is infrequently achieved. Previously, an inverse trend was most evident among the non-sham controlled randomized controls, predominantly noted in the DENERVHTA trial^19^ and the Oslo RDN trial^17^. However, the newer trials^8–10^ have shown a trend towards improved blood pressure control with renal denervation in comparison to medical therapy alone.

Significant heterogeneity was observed when all the included studies were analyzed together. The differences in blood pressure changes could in part be explained by differences in study design, type of control group and type of RDN procedure performed. It is widely believed that the experience of an operator plays a major role in the success of the procedure. In the initial trials, patients were enrolled from centers with very low operator volume which probably affected the success of the procedure. However over a period of time, since the first trial (Symplicity HTN 2^16^) was performed in 2010, operators have become more proficient with the procedure leading to better outcomes. This also explains the heterogeneity of the results among the trials. With the exception of the change in 24 hr. systolic blood pressure, subgroup analysis (Sham vs No sham) and meta-regression analysis did not change the significance of blood pressure and renal function outcomes between RDN and control groups. The discrepancies observed between changes in 24 hr. blood pressure and office blood pressure between the two groups could be explained by “regression to the mean” effect, which occurs as a result of using specified office blood pressure thresholds while enrolling patients in these trials^25^. Sham control studies allow accounting for potential confounding factors including “regression to the mean effect”, the placebo effect of the procedure and improved medication adherence after renal denervation.

Most trials included in our analysis showed an improvement in blood pressure in the control group during the course of the trial. This could be attributed to the following reasons (1) Placebo effect in patients who underwent sham controlled procedures. (2) Improvement in adherence to medications as all the patients in the trials was monitored using medication adherence tools such as witnessed medication intake, maintenance of diary, assessment of plasma drug concentration, etc. and (3) Recruiting patients who weren’t maximized on medical therapy.

The Symplicity Catheter system (Ardian, Mountain View, CA, USA) is commonly used in contemporary practice. However it has several limitations including (1) presence of single electrode leading to longer ablation time, (2) presence of unipolar electrode makes a selection of ablation site challenging and (3) inability to ablate deep renal sympathetic nerve due to low radiofrequency power of the catheter limiting the penetration depth. To overcome these limitations, Symplicity Spyral catheter system (Medtronic, Galway, Ireland) and EnligHTN catheter system (St. Jude Medical, Saint Paul, MN) were introduced. They are multi-electrode catheters containing 4 electrodes and can simultaneously ablate 4 locations at the same time. Results from SPYRAL HTN-OFF MED and SPYRAL HTN-ON MED^8^ trials showed a significant reduction in blood pressure with RDN. The use of multiple catheter systems could potentially contribute to the heterogeneity noted in our analysis.

The adjusted pooled analysis of the 5 sham controlled studies showed a modest but significant benefit in the mean difference in 24 hr. systolic blood pressure change at 6 months which is contrary to the prior meta-analysis published^26^. The significance was predominantly driven by SPYRAL-HTN-ON MED trial^8^. The renal denervation procedure (using RF ablation) performed in this trial targeted branch vessels beyond the proximal main renal artery which was unique. Animal studies have shown a greater reduction in renal norepinephrine levels with RF ablation if the distal extra-renal artery branches were targeted^27^. This hypothesis was further tested by Petrov *et al*. in a non-randomized study (N = 119) which compared the efficacy of blood pressure reduction comparing conventional renal artery denervation strategy to a novel renal denervation strategy that targets the distal branches of the renal artery in addition to the proximal renal artery. They showed a greater decrease in office and 24 hr. systolic and diastolic blood pressure among the patients who underwent the novel procedure compared to the conventional procedure. This technique of radiofrequency RDN may explain the predominantly greater reduction in blood pressure noted in the RDN group in the SPYRAL-HTN-ON MED trial.

RADIOSOUND-HTN trial^28^ was performed to test head to head comparison between US ablation, conventional RF ablation of main renal arteries and RF ablation of main + side branches of renal arteries. The study showed that significantly higher reduction in ambulatory blood pressure with US ablation compared to conventional RF ablation of main renal arteries. However, no difference in blood pressure reduction was noted between patients who underwent US ablation compared to patients who underwent RF ablation of main and side branches of renal arteries. This further explains the degree of heterogeneity noted in our analysis.

A predominant confounder in randomized control trials evaluating the efficacy of a novel therapy is the medication adherence among study participants. In this instance, if the medication adherence significantly improves after the patient undergoes renal denervation; the true effect of the procedure would be difficult to estimate. Among the 15 included studies, only four studies used robust methods to assess medication adherence using witnessed medication intake^17^ or plasma drug concentration^8,15,20^ for a total of 344 patients (approximately 24% of the total study population). The remaining 11 studies assessed medication adherence using compliance diary or validated questionnaires. Medication non-adherence is noted among 50% of hypertensive patients and its prevalence increases especially in patients with RH. Accurate assessment of medication non-adherence is difficult given the invasive nature of the tests and incorporation into routine practice remains a challenge given the tests are cost-intensive.

We have also shown that patients who underwent RDN had no significant changes in renal function (eGFR) compared to the medical therapy group at 6 months post-renal denervation, supporting the safety profile of the procedure. Our findings are consistent with prior published studies^6,29^. The positive results noted in the newer trials did not affect the overall result of the meta-analysis.

## Limitations

The meta-analysis reported here combines data across studies in order to estimate treatment effects with more precision than is possible in a single study. Limitations include incorporating studies with different control arms and designs to increase the study population and maximizing the likelihood of estimating a treatment effect. However, the results of the subgroup analyses would be considered more meaningful given the lower heterogeneity. This specifically applies to the pooled analysis of 5 studies which showed a modest improvement in 24 systolic blood pressures with renal denervation. Publication bias might account for some of the effects we observed. Smaller trials are, in general, analyzed with less methodological rigor than larger studies, and an asymmetrical funnel plot suggests that selective reporting may have led to an overestimation of effect sizes in small trials. The lack of individual participant level data for meta-analysis and subgroup analysis is a limitation of our study. Given the nature of meta-analysis, the inherent weakness of the individual studies will be inherited in our study. The variability of expertise among all the physicians performing renal denervation could potentially affect the outcome of the procedure, thereby the overall outcome of the individual trials.

## Materials and Methods

### Data sources and searches

An extensive electronic search of Embase, Scopus, Web of Science, PubMed, and manual review of the literature was performed for relevant articles using the following search terms: “renal denervation”, “renal sympathetic denervation”, and “resistant hypertension”. The search was restricted to publications in English and the final search was performed till from Jan 2008 to June 2018 (Supplementary data).

### Study selection

Studies were included if they satisfied the following criteria: (1) Age of the study population >18 yrs.; (2) Original randomized control trials comparing renal denervation with medical therapy ± sham procedure, (3) Published a detailed study protocol, (4) Follow-up duration of ≥6months, (5) Patient population diagnosed with resistant hypertension on ≥3 blood pressure medications, (6) Reported estimated glomerular filtration rate (eGFR), average office or ambulatory systolic and diastolic blood pressure at baseline and 6 months following intervention, and (7) Reported characteristics of the overall study population. Studies were excluded in case of partial/complete overlap of the study population with a study already included in the analysis and also in cases of incomplete or incorrect reporting of data. All eligible studies complied with Consolidated Standards of Reporting Trials (CONSORT) quality criteria^30^.

### Data extraction and quality assessment

The initial search was performed by two reviewers (PA; JS), and studies were selected for inclusion by mutual consensus. In case of disagreement, a third reviewer (RA) resolved disagreements through discussion to achieve a consensus. Once the studies were selected for inclusion, the data were screened to meet the inclusion criteria and thereafter the following data points were extracted: number of anti-hypertensive medications, class of anti-hypertensive medications used, eGFR, average office or ambulatory systolic and diastolic blood pressure at baseline and 6 months following the intervention. Additionally, cumulative demographic and baseline characteristics of the patient population included in the study were also extracted. The quality of included studies was assessed using the Jadad scale for randomized control trials^31^. When both intention to treat and per protocol outcomes were reported by the authors, we only considered intention to treat values for our analysis. We directly contacted the authors when additional information was missing from the initial studies.

### Data synthesis and Analysis

Continuous variables are presented as mean and standard deviation and categorical data as percentages. The aim of our analysis was to assess the change in blood pressure (24 hr. and office measured) and renal function in patients who underwent renal denervation in comparison to patients on optimal medical therapy. We calculated an unadjusted pooled change in eGFR, office and 24 hr. blood pressure in both renal denervation and control groups. We calculated a weighted standardized mean difference among outcomes between the renal denervation group and control group with random effects models (DerSimonian and Laird). Statistical heterogeneity was defined as I^2^ statistic value greater than 50%. Publication bias was visually assessed using Deek’s funnel plot followed by a trim-and-fill procedure. The Deek’s funnel plot utilizes regression of diagnostic log odd’s ratio against 1/square root (effective sample size) and further weighting by effective sample size. A p-value of the slope coefficient of <0.10 indicated asymmetry and thereby publication bias. The significance of asymmetry in the funnel plot will be assessed using Eggers test and a trim-and-fill test will be performed if applicable to correct asymmetry^32,33^. We performed a sensitivity analysis by type of control arm (sham vs no sham controlled).

Meta-regression analysis was performed to identify potential sources of heterogeneity. We performed meta-regression for covariates including sample size (less or more than 100), region of study, year of publication, quality of study, sham vs no sham controlled, age, gender, body mass index, proportion of patients with hypertension, coronary artery disease and diabetes mellitus to identify potential sources of heterogeneity. This meta-analysis was performed in compliance with Preferred Reporting Items for Systematic reviews and Meta-Analyses (PRISMA) guidelines^34^. All the above analyses were performed in R, version 3.4.3, using “mada” package.

## Conclusion

Our meta-analysis of 15 randomized control trials showed no significant benefit of renal denervation on blood pressure control in patients with resistant hypertension. Subgroup analysis of sham control studies showed a modest benefit in 24 hr. systolic blood pressure at 6 months with renal denervation. Our analysis substantiates the safety profile of the procedure o based on renal function at 6 months post procedure. Given the evidence of procedure-related factors, objective assessment of drug adherence, novel renal denervation techniques, and sham control study design affects the overall study outcomes; future clinical trials must account for these variances in order to better assess the effectiveness of renal denervation in patients with RH. Furthermore, the type of ablation therapy used and target sites for ablation (conventional versus branch renal artery ablation techniques) must be explored in future trials. Based on the current evidence, patients with resistant hypertension and no identifiable secondary cause (renovascular or renal parenchymal disease, etc.), maximized on lifestyle interventions and medical therapy by a hypertension specialist may benefit from renal denervation with an experienced operator.

## Supplementary information

Supplementary Data
