# Supplementary material for: Renal Denervation for Resistant Hypertension in the contemporary era: A Systematic Review and Meta-analysis
Source: Sci Rep. 2019 Apr 17;9:6200. doi: 10.1038/s41598-019-42695-9 (PMC6470219; doi:10.1038/s41598-019-42695-9)
Supplement: Supplementary file 1 — Supplementary Data [file 41598_2019_42695_MOESM1_ESM.docx]

**Supplementary data**

**Renal Denervation for Resistant Hypertension in the contemporary era:
A Systematic Review and Meta-analysis**

Pradyumna Agasthi M.D^1^, Justin Shipman M.D ^1^, Reza Arsanjani M.D^1^ , Moses Ashukem M.D^2^, Marlene. E. Girardo M.S^3^, Charan Yerasi M.D^4^, Nithin. R. Venepally M.B.B.S^1^, Floyd David Fortuin M.D^1^, Farouk Mookadam M.B.B.Ch^1^

**Author affiliations:**

1. Division of Cardiovascular Diseases, Mayo Clinic Arizona, Phoenix, Arizona
2. Division of Cardiovascular Medicine, University of Arizona, Phoenix, Arizona
3. Department of Health Sciences Research, Mayo Clinic Arizona, Scottsdale, Arizona
4. Department of Cardiovascular Diseases, St. Joseph’s Hospital and Medical Center, Phoenix, Arizona

**Competing Interests:** The author(s) declare no competing interests.

**Corresponding Author:** Pradyumna Agasthi, M.D

**Address of Correspondence:** 5777 E Mayo Blvd, Phoenix, AZ 85054

**Phone Number:** 4803014072

**E-mail:** pradyumna_agasthi@hotmail.com

**Supplementary data:**

S1. Baseline characteristics of patients (Part 1).

S2. Baseline characteristics of patients (Part 2).

S3. Quality of randomized clinical trials assessed using Jadad scale.

S4. Mean change in office and 24 hr. blood pressures; and estimated glomerular filtration rate from baseline to 6 months post randomization.

S5. Meta-regression analysis for office and 24 hr. blood pressure; and estimated glomerular filtration rate.

| Author | Study Acronym | Sample size >100 or <100 | SHAM control | Age yrs (mean±SD) | | Female Sex % | | Race white % | | BMI kg/m2 mean±SD | | DM2 % | | CAD % | | Hypercholestrolemia % | |
| --- | --- | --- | --- | --- | --- | --- | --- | --- | --- | --- | --- | --- | --- | --- | --- | --- | --- |
|  |  |  | Yes or No | RDN | control | RDN | control | RDN | control | RDN | control | RDN | control | RDN | control | RDN | control |
| Esler et al | Symplicity HTN 2 | >100 | No | 59±11.5 | 58.1±13 | 32.7 | 60 | 98 | 97.1 | 30.8±5.2 | 31.5±5.3 | 42.9 | 28.6 | 18.4 | 5.7 | 53.1 | 45.7 |
| Bhatt et al | Symplicity HTN 3 | >100 | Yes | 57.9±10.4 | 56.2±11.2 | 40.9 | 35.7 | 73 | 69.6 | 34.2±6.5 | 33.9±6.4 | 47 | 40.9 | 27.7 | 25.1 | 69.2 | 64.9 |
| Fadl Elmula et al | OSLO RDN | <100 | No | 57±10.9 | 62.7±5.1 | 22 | 0 | 100 | 100 | 29±5.3 | 30±5.3 | 22 | 30 | 11 | 60 | 22 | 40 |
| Rosa et al | PRAGUE-15 | >100 | No | 56±12 | 59±9 | 23 | 37 | 100 | 100 | 31.2±4.3 | 33.4±4.7 | 22 | 17 | 6 | 7 | - | - |
| Azizi et al | DENERHTN | >100 | No | 55.2±10.8 | 55.2±10.1 | 35.8 | 39.6 | 79.2 | 77.4 | 30.7±4.8 | 29.7±4.5 | 17 | 26.4 | 30.2 | 20.8 | 52.8 | 39.6 |
| Desch et al | Symplicity-Flex | <100 | Yes | 64.5±7.6 | 57.4±8.6 | 23 | 31 | - | - | 31.9±4.4 | 31.2±4.6 | 54 | 36 | 60 | 47 | - | - |
| Schneider et al | ISAR-denerve | <100 | No | 62±9 | 60±8 | 22 | 11 | 89 | 89 | 31±6 | 33±8 | 89 | 33 | 67 | 56 | 67 | 67 |
| Kario et al | Symplicity HTN-Japan | <100 | No | 59.5±11.9 | 56±13 | 31.8 | 15.8 | 0 | 0 | 27±5.5 | 28±3.9 | 36.4 | 63.2 | - | - | 27.3 | 36.8 |
| Mathiassen et al | ReSET | <100 | Yes | 54.3±7.8 | 57.1±9.6 | 25 | 27 | 97 | 97 | 28.2±5 | 28.8±3.9 | 25 | 31 | 6 | 15 | - | - |
| Oliveras et al | DENERVHTA | <100 | No | 61.9±6.6 | 64.9±8.2 | 45 | 31 | 100 | 85 | 33.7±7.4 | 30.6±3.6 | 36 | 62 | - | - | 100 | 85 |
| de Jager et al | Sympathy | >100 | No | 62±12 | 60±10 | 57.9 | 70.5 | 96.8 | 95.5 | 28.6±4.8 | 29.4±4.6 | 27.4 | 31.8 | 43.2 | 43.2 | - | - |
| Jacobs et al | INSPiRED | <100 | No | 48.4±10.8 | 47.9±8.8 | 50 | 55.6 | - | - | 29.3±4.5 | 31±4.9 | 0 | 33.3 | - | - | 66.7 | 44.4 |
| Schmeider et al | WAVE-IV | <100 | Yes | 60.3±11.2 | 62±11.1 | 18.6 | 35.9 | - | - | 29.9±4.5 | 29.8±4.2 | 27 | 26 | 4.8 | 0 | 4.8 | 0 |
| Warchol-Celinska et al | No Acronym | <100 | No | 55.9±9.4 | 54.5±9.2 | 20 | 20 | - | - | 34±6.2 | 34.7±4.5 | 47 | 30 | 40 | 23 | 93 | 93 |
| Kandazari et al | SPYRAL HTN-ON MED | <100 | Yes | 53.9±8.7 | 53±10.7 | 13 | 19 | 34 | 36 | 31.4±6.4 | 32.5±4.6 | 13 | 19 | 3 | 2 | - | - |

**Table S1:** Baseline Characteristics of patients (Part 1).

Yrs = years, SD = standard deviation, RDN = renal denervation, BMI = body mass index, DM2 = diabetes mellitus type II, CAD = Coronary artery disease.

| Author | study acronym | number of anti-htn meds RDN | | number of anti-htn meds control | | ACE/ARB % | | direct renin inhibitors % | | beta blockers % | | CCB % | | diuretics % | | aldosterone antagonists % | | vasodilators % | | alpha blockers % | | centrally acting sympatholytics % | |
| --- | --- | --- | --- | --- | --- | --- | --- | --- | --- | --- | --- | --- | --- | --- | --- | --- | --- | --- | --- | --- | --- | --- | --- |
|  |  | mean | SD | Mean | SD | RDN | control | RDN | control | RDN | control | RDN | control | rdn | control | RDN | control | RDN | control | RDN | control | RDN | control |
| Esler et al | Symplicity HTN 2 | 5.3 | - | 5.2 | - | 100 | 82.9 | 16.3 | 22.9 | 81.6 | 62.9 | 77.6 | 77.1 | 18.4 | 22.9 | 184 | 22.9 | 8.2 | 2.9 | 8.2 | 2.9 | 51 | 42.9 |
| Bhatt et al | Symplicity HTN 3 | 5.1 | 1.4 | 5.2 | 1.4 | 99.2 | 94.7 | 7.1 | 7 | 85.2 | 86 | 69.8 | 73.1 | 22.5 | 28.7 | 22.5 | 28.7 | 36.8 | 45 | 11 | 13.5 | 49.2 | 43.9 |
| Fadl Elmula et al | OSLO RDN | 5.1 | 1.6 | 5 | 1.2 | 100 | 100 | 22 | 0 | 56 | 90 | 89 | 70 | 33 | 60 | 33 | 60 | 0 | 20 | 56 | 20 | 56 | 40 |
| Rosa et al | PRAGUE-15 | 5.1 | 1.2 | 5.4 | 1.2 | 100 | 100 | 0 | 0 | 66 | 6.9 | 89 | 89 | 27 | 24 | 27 | 24 | 0 | 0 | 54 | 46 | 54 | 61 |
| Azizi et al | DENERHTN | 3 | - | 3 | - | 100 | 100 | 0 | 0 | 0 | 0 | 100 | 100 | 0 | 0 | 0 | 0 | 0 | 0 | 0 | 0 | 0 | 0 |
| Desch et al | Symplicity-Flex | 4.4 | 1.3 | 4.3 | 1.3 | 97 | 100 | 3 | 8 | 91 | 94 | 69 | 64 | 3 | 6 | 3 | 6 | 6 | 11 | 21 | 14 | 26 | 28 |
| Schneider et al | ISAR-denerve | 5.1 | 1.3 | 4.3 | 0.5 | 56 | 88 | 0 | 0 | 78 | 89 | 89 | 100 | 0 | 0 | 0 | 0 | 33 | 0 | 56 | 44 | 78 | 22 |
| Kario et al | Symplicity HTN-Japan | 4.9 | 1.5 | 4.9 | 1.8 | 100 | 100 | 0 | 0 | 81.8 | 68.4 | 95.5 | 94.7 | 45.5 | 36.8 | 45.5 | 36.8 | 0 | 0 | 22.7 | 42.1 | 0 | 0 |
| Mathiassen et al | ReSET | 4.1 | 1.2 | 4.2 | 1.1 | 100 | 100 | 3 | 6 | 81 | 76 | 53 | 85 | 22 | 21 | 22 | 21 | 17 | 6 | 11 | 21 | 17 | 15 |
| Oliveras et al | DENERVHTA | 4.3 | 0.8 | 3.9 | 0.6 | 100 | 92 | 0 | 0 | 55 | 77 | 91 | 69 | 0 | 0 | 0 | 0 | 0 | 0 | 55 | 39 | 18 | 8 |
| de Jager et al | Sympathy | 3.7 | 1.5 | 3.4 | 1.5 | 86.3 | 93.2 | 3.2 | 0 | 63.2 | 59.1 | 63.2 | 61.4 | 5.3 | 6.8 | 5.3 | 6.8 | 0 | 0 | 31.6 | 25 | 9.5 | 6.8 |
| Jacobs et al | INSPiRED | - | - | - | - | 100 | 100 | 0 | 0 | 66.7 | 77.8 | 100 | 88.9 | 83.3 | 55.6 | 83.3 | 55.6 | 0 | 0 | 0 | 0 | 0 | 0 |
| Schmeider et al | WAVE-IV | 4.3 | 1.4 | 5 | 1.5 | 100 | 97.4 | 0 | 0 | 59.5 | 58.9 | 80.9 | 82.1 | 18.1 | 13 | 18.1 | 13 | 0 | 0 | 0 | 0 | 0 | 0 |
| Warchol-Celinska et al | No Acronym | 5 | 0.3125 | 5 | 0.25 | 100 | 100 | 0 | 0 | 93 | 90 | 90 | 90 | 27 | 10 | 27 | 10 | 0 | 0 | 57 | 70 | 27 | 10 |
| Kandazari et al | SPYRAL HTN-ON MED | 2.2 | 0.9 | 2.3 | 0.8 | 82 | 83 | 0 | 0 | 11 | 14 | 71 | 74 | 0 | 0 | 0 | 0 | 0 | 0 | 0 | 0 | 0 | 0 |

**Table S2:** Baseline Characteristics of patients (Part 2).

Anti-HTN meds = anti-hypertensive medications, RDN = renal denervation, SD = standard deviation, ACE = angiotensin converting enzyme inhibitor, CCB = calcium channel blocker.

| Study | Was the study described as random? | Was the randomization scheme described and appropriate? | Was the study described as double-blind? | Was the method of double blinding appropriate? | Was there a description of dropouts and withdrawals? | Score |
| --- | --- | --- | --- | --- | --- | --- |
| DENERHTN trial | 1 | 1 | 0 | N/A | 1 | 3 |
| DENERVHTA trial | 1 | 1 | 0 | N/A | 1 | 3 |
| Symplicity-Flex trial | 1 | 1 | 1 | 1 | 1 | 5 |
| OSLO RDN trial | 1 | 1 | 0 | N/A | 1 | 3 |
| ISAR-denerve trial | 1 | 1 | 0 | N/A | 1 | 3 |
| INSPiRED trial | 1 | 1 | 0 | N/A | 1 | 3 |
| PRAGUE-15 trial | 1 | 1 | 0 | N/A | 1 | 3 |
| ReSET trial | 1 | 1 | 1 | 1 | 1 | 5 |
| SPYRAL HTN-ON MED trial | 1 | 1 | 1 | 1 | 1 | 5 |
| Sympathy trial | 1 | 1 | 0 | N/A | 1 | 3 |
| Symplicity HTN 2 trial | 1 | 1 | 0 | N/A | 1 | 3 |
| Symplicity HTN 3 trial | 1 | 1 | 1 | 1 | 1 | 5 |
| Symplicity HTN-Japan trial | 1 | 1 | 0 | N/A | 1 | 3 |
| Warchol-Celinska et al | 1 | 1 | 0 | N/A | 0 | 2 |
| WAVE-IV trial | 1 | 1 | 1 | 1 | 1 | 5 |

**Table S3**: Quality of randomized clinical trials assessed using Jadad scale.

| Author | study acronym | change in SBP (office) at 6 months mmHg (mean ± SD) | | change in SBP (24 hr) at 6 months mmHg (mean ± SD) | | change in DBP (office) at 6 months mmHg (mean ± SD) | | change in DBP (24 hr) at 6 months mmHg (mean ± SD) | | change in eGFR at 6 months (mL/min/1.73 m2) (mean ± SD) | |
| --- | --- | --- | --- | --- | --- | --- | --- | --- | --- | --- | --- |
|  |  | RDN | control | RDN | control | RDN | control | RDN | control | RDN | control |
| Esler et al | Symplicity HTN 2 | -31.7 ± 23.1 | -23.7 ± 27.5 |  |  | -11.7 ± 11.2 | -8.4 ± 12.1 |  |  | 0.2 ± 11 | 0.9 ± 12 |
| Bhatt et al | Symplicity HTN 3 | -14.13 ± 23.93 | -11.74 ± 25.94 | -6.75 ± 15.11 | -4.79 ± 17.2 | -6.6 ± 11.9 | -4.6 ± 13.6 | -4.1 ± 9.2 | -3.1 ± 10.1 | -2.12 ± 12.91 | -1.72 ± 12.14 |
| Fadl Elmula et al | OSLO RDN | -8 ± 15 | -28 ± 13 | -10 ± 9 | -19 ± 11 | -2 ± 8 | -11 ± 8 | -7 ± 6 | -11 ± 8 | 1.8 ± 8 | -4.7 ± 6.5 |
| Rosa et al | PRAGUE-15 | -12.4 ± 16.5 | -14.3 ± 19.78 | -8.6 ± 11.85 | -8.1 ± 17.22 | -7.4 ± 12.57 | -7.3 ± 10.99 | -5.7 ± 8.26 | -4.5 ± 8.06 | 1.5 ± 11.4 | -3.1 ± 13.8 |
| Azizi et al | DENERHTN | -15.1 ± 19.28 | -9.5 ± 18.25 | -15.4 ± 12.74 | -9.5 ± 12.05 | -9.1 ± 10.33 | -6 ± 10.33 | -9.7 ± 7.92 | -6.6 ± 7.58 | -4.9 ± 9.5 | -5.3 ± 12.8 |
| Desch et al | Symplicity-Flex |  |  | -7 ± 10.54 | -3.5 ± 9.61 |  |  | -2.8 ± 5.27 | -2.1 ± 5.53 | -2.8 ± 10.9 | -0.01 ± 10.9 |
| Schneider et al | ISAR-denerve | -23 ± 14.5 | 1 ± 13 | -2.88 ± 11.1 | -5 ± 11.1 | -9 ± 8.7 | -1 ± 10.1 | -1.38 ± 6 | -4.67 ± 9.6 | -2.15 ± 2.8 | -1.37 ± 2.8 |
| Kario et al | Symplicity HTN-Japan | -16.6 ± 18.5 | -7.9 ± 21 | -7.5 ± 12 | -1.4 ± 10.2 | -5.9 ± 11.1 | -1 ± 8.8 | -4.2 ± 7.4 | -0.4 ± 6.7 | 1.86 ± 9.7 | -3.81 ± 9.4 |
| Mathiassen et al | ReSET |  |  | -3.7 ± 16.4 | -2.6 ± 12.8 |  |  | -1.7 ± 8.6 | -2.6 ± 7.5 |  |  |
| Oliveras et al | DENERVHTA | -17.5 ± 18.45 | -29.4 ± 18.7 | -5.7 ± 13.54 | -23.6 ± 13.74 | -7.5 ± 11.91 | -12.7 ± 11.92 | -3.7 ± 6.85 | -10.2 ± 6.78 | -3 ± 10.27 | -13.7 ± 10.42 |
| de Jager et al | Sympathy | -7.5 ± 24.54 | 0.7 ± 25 | -5.6 ± 23.07 | -6.6 ± 21.05 | -4.4 ± 14.73 | 0.9 ± 15.46 | -3.5 ± 13.25 | -3.9 ± 12.5 |  |  |
| Jacobs et al | INSPiRED | -11.9 ± 17.63 | 7.6 ± 23.02 | -21.7 ± 11.24 | 0.7 ± 20.42 | -8.2 ± 8.86 | 2.2 ± 15.87 | -12.8 ± 9.91 | 0.3 ± 11.84 | -1.1 ± 8.23 | 1.5 ± 11.31 |
| Schmeider et al | WAVE-IV | -12.8 ± 26 | -23 ± 20 | -7.11 ± 13 | -5.9 ± 15 | -5.1 ± 15 | -8.9 ± 12 | -5 ± 9.9 | -4.5 ± 9.5 | -1.8 ± 8.8 | -3.1 ± 7.7 |
| Warchol-Celinska et al | No Acronym | -24 ± 18 | -3 ± 12 | -10 ± 4.14 | -3 ± 4.42 | -11 ± 3.1 | -3 ± 2.48 | -7 ± 2.71 | -3 ± 2.46 |  |  |
| Kandazari et al | SPYRAL HTN-ON MED | -9.4 ± 12.47 | -2.6 ± 13.48 | -9 ± 11.25 | -1.6 ± 11.55 | -5.2 ± 7.61 | -1.7 ± 8.34 | -6 ± 7.61 | -1.9 ± 8.98 |  |  |

**Table S4:** Mean change in office and 24 hr blood pressures; and estimated glomerular filtration rate from baseline to 6 months post randomization.

SBP = systolic blood pressure, mmHg = millimeters of mercury, SD = standard deviation, RDN = renal devervation, DBP = diastolic blood pressure, eGFR = estimated glomerular filtration rate.

| Meta-regression analysis: Office SBP | | | |
| --- | --- | --- | --- |
|  | Meta-regression coefficient | 95% CI | p-value |
| Year of Publication | -0.0854 | (-0.2210, 0.0502) | 0.2170 |
| Sample Size | 0.2400 | (-0.1742, 0.6543) | 0.2561 |
| Location | -0.4141 | (-1.1856, 0.3574) | 0.2928 |
| SHAM | 0.5850 | (0.0011, 1.1689) | 0.0496 |
| Age (diff) | -0.1182 | -0.2537, 0.0172) | 0.0871 |
| BMI (diff) | 0.0578 | (-0.1423, 0.2579) | 0.5713 |
| CAD (diff) | -0.0223 | (-0.0407, -0.0039) | 0.0177 |
| Meta-regression analysis 24-hr SBP | | | |
| Year of Publication | -0.1002 | (-0.3052, 0.1048) | 0.3380 |
| Sample Size | 0.3488 | (-0.4026, 1.1001) | 0.3629 |
| Location | -0.3802 | (-1.2437, 0.4833) | 0.3881 |
| SHAM | 0.3909 | (-0.4407, 1.2225) | 0.3569 |
| Age (diff) | 0.0406 | (-0.0246, 0.1057) | 0.2224 |
| BMI (diff) | -0.0668 | (-0.2855, 0.1520) | 0.5497 |
| CAD (diff) | -0.0343 | (-0.0548, -0.0138) | 0.0010 |
| Sex (diff) | -0.0119 | (-0.0481, 0.0242) | 0.5174 |
| Meta-regression analysis Office DBP | | | |
| Year of Publication | -0.1807 | (-0.3725, 0.0111) | 0.0649 |
| Sample Size | 0.5375 | (-0.0817, 1.1567) | 0.0889 |
| Location | -0.7971 | (-1.8956, 0.3013) | 0.1549 |
| SHAM | 1.2325 | (0.2836, 2.1814) | 0.0109 |
| Age (diff) | -0.0478 | (-0.2490, 0.1535) | 0.6417 |
| BMI (diff) | 0.0695 | (-0.3719, 0.2329) | 0.6526 |
| CAD (diff) | -0.0305 | (-0.0554, -0.0056) | 0.0164 |
| Meta-regression analysis 24-hr DBP | | | |
| Year of Publication | -0.0676 | (-0.2718, 0.1365) | 0.5161 |
| Sample Size | 0.3531 | (-0.3932, 1.0994) | 0.3537 |
| Location | -0.5143 | (-1.3732, 0.3447) | 0.2406 |
| SHAM | 0.5482 | (-0.2781, 1.3745) | 0.1935 |
| Age (diff) | 0.0471 | (-0.0179, 0.1120) | 0.1554 |
| BMI (diff) | -0.0723 | (-0.2903, 0.1457) | 0.5157 |
| CAD (diff) | -0.0289 | (-0.0491, -0.0087) | 0.0050 |
| Sex (diff) | -0.0061 | (-0.0421, 0.0299) | 0.7407 |

**Table S5:** Meta-regression analysis for office and 24 hr blood pressure; and estimated glomerular filtration rate.

SBP = systolic blood pressure, CI = confidence interval, BMI = body mass index, CAD = coronary artery disease, DBP = diastolic blood pressure.

**Search strategy:**

1. **6.15.18 PubMed Search Strategy**: (((("high blood pressure") OR hypertension) OR "Hypertension"[Mesh])) AND (((renal denervation) OR (("Denervation"[Mesh]) AND (kidney OR renal))) OR "Kidney/innervation"[Mesh]) Filters: Randomized Controlled Trial (57)
2. **6.21.18 Embase <1988 to 2018 Week 25> Search Strategy**:
3. high blood pressure.mp. or hypertension/ OR hypertension.mp. AND (renal denervation.mp. or kidney denervation/) or (denervation/ and (kidney or renal).mp. [mp=title, abstract, heading word, drug trade name, original title, device manufacturer, drug manufacturer, device trade name, keyword, floating subheading word, candidate term word]) OR kidney innervation/ limit 10 to randomized controlled trial (136)
4. **6.21.18 Cochrane Database of Systematic Reviews <2005 to June 20, 2018> Search Strategy**:
5. high blood pressure.mp. [mp=title, abstract, full text, keywords, caption text] or hypertension.mp. [mp=title, abstract, full text, keywords, caption text] AND renal denervation.mp. [mp=title, abstract, full text, keywords, caption text] AND (RCT or randomized controlled trial).mp. [mp=title, abstract, full text, keywords, caption text] (1)
6. **6.21.18 Scopus Search Strategy**: ( TITLE-ABS-KEY ( {high blood pressure} OR hypertension ) AND TITLE-ABS-KEY ( {renal denervation} OR {kidney denervation} ) AND TITLE-ABS-KEY ( rct OR {randomized controlled trial} ) )
7. **6.21.18 Web of Science Search Strategy**: **TOPIC:** (hypertension OR "high blood pressure") AND TOPIC: ("renal denervation" OR "kidney denervation") AND TOPIC: (RCT OR "randomized controlled trial") Timespan: All years. Indexes: SCI-EXPANDED, ESCI.

| DATABASE | RESULTS | DUPLICATES | REMAINING |
| --- | --- | --- | --- |
| PubMed | 57 | 36 | 21 |
| Embase | 136 | 14 | 122 |
| Scopus | 331 | 84 | 247 |
| Web of Science | 83 | 17 | 66 |
| Cochrane Database of Systematic Reviews | 1 | 1 | 0 |
| **TOTAL** | 608 | 152 | 456 |
